# A Comparison of Medial and Lateral Ligament Balance in Functional Versus Simulated Mechanical Alignment in Total Knee Arthroplasty Across CPAK Phenotypes: A Single-Center Study

**DOI:** 10.3390/jcm15176812

**Published:** 2026-09-02

**Authors:** Anne Ruth van Meijeren, Daniëlle Langeloo, Hans-Peter van Jonbergen, Martin Stevens, Marrigje Floor Meijer

**Affiliations:** 1 Department of Surgery, Martini Ziekenhuis Groningen, 9728 NT Groningen, The Netherlands; 2 Department of Orthopaedic Surgery, Deventer Hospital, 7416 SE Deventer, The Netherlands; 3 Department of Orthopaedic Surgery, University Medical Center Groningen, 9713 GZ Groningen, The Netherlands; m.stevens@umcg.nl; 4 Division of Orthopaedics at Campus Pius-Hospital, School of Medicine and Health Sciences, Carl von Ossietzky Universität, 26129 Oldenburg, Germany; 5 Department of Orthopaedic Surgery, Bergman Clinics Arnhem, 6842 CV Arnhem, The Netherlands

**Keywords:** functional alignment, mechanical alignment, robotic-assisted total knee arthroplasty, soft tissue balance

## Abstract

**Background/Objectives**: Despite the excellent long-term outcomes of total knee arthroplasty (TKA), up to 10% of patients remain dissatisfied. Robotic-assisted TKA enables individualized alignment strategies, such as functional alignment (FA). Proper alignment and soft tissue balance are considered key factors for positive clinical outcomes. This study compared medial and lateral ligament balance between simulated mechanical alignment (MA) and FA across different Coronal Plane Alignment of the Knee (CPAK) types using intraoperative robotic simulations. **Methods**: In this observational cohort study, 200 patients undergoing primary robotic-assisted TKA were included. Knee laxity was assessed intraoperatively under simulated MA and FA within the same knee. Laxity was defined as medial and lateral joint space opening under varus and valgus stress in flexion and extension. Balance was defined as an absolute difference of ≤2 mm. Outcomes were stratified by native CPAK phenotype in an exploratory subgroup analysis. **Results**: Functional alignment resulted in significantly improved ligament balance compared to simulated MA across CPAK types. The largest improvements were observed in CPAK type II, with the proportion of overall balanced knees increasing from 7.7% in simulated MA to 88.5% in FA. Improvements were observed in CPAK types II, V, VI and VIII and were mainly driven by a reduction in extension imbalance. **Conclusions**: Functional alignment leads to improved medial and lateral ligament balance compared to simulated MA. These findings suggest that the effect of the alignment strategy on soft tissue balance may differ according to the underlying knee phenotype.

## 1. Introduction

Total knee arthroplasty (TKA) is a successful treatment for end-stage osteoarthritis. Although the majority of patients are satisfied after knee replacement, 10% of patients remain dissatisfied with their outcomes [1]. The underlying reasons are not fully understood. Proper alignment and soft tissue balancing are considered essential for good clinical outcomes, prosthesis survival and patient satisfaction after TKA [2,3]. Robotic-assisted total knee arthroplasty (TKA) facilitates the implementation of individualized alignment strategies by enabling accurate preoperative planning and intraoperative assessment of ligament balance [4,5].

Mechanical alignment (MA) has been the gold standard for knee prosthesis positioning, with the aim of restoring neutral limb alignment. However, this approach does not account for individual anatomic variation and may result in ligament imbalance, potentially requiring additional soft tissue releases [6]. This may be particularly relevant in patients with a constitutional varus knee alignment [6].

Novel alignment strategies such as functional alignment (FA) and kinematic alignment (KA) are aimed at individualized prosthetic alignment based on patient-specific anatomy and ligament laxity. FA is an individualized alignment strategy that aims to restore the native coronal alignment and joint line orientation while achieving ligament balance through patient-specific implant positioning within predefined boundaries [5,7]. Unlike MA, FA does not aim to restore a neutral mechanical axis in every patient; it adapts implant positioning to the patient’s individual anatomy and soft tissue laxity. This approach may reduce the need for soft tissue releases compared with conventional alignment strategies [5,7,8].

**Figure 1:**
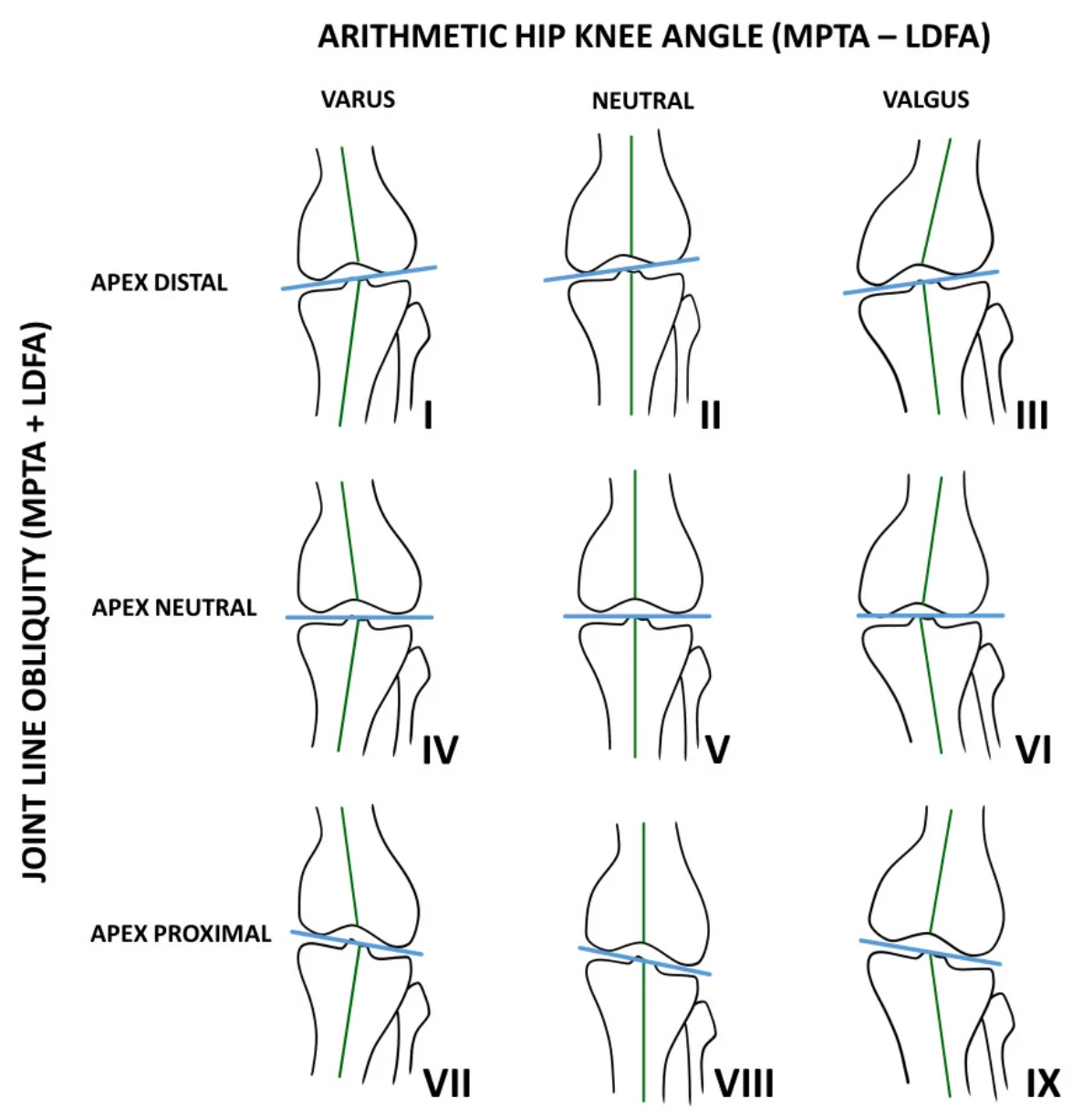
Coronal Plane Alignment of the Knee (CPAK) classification matrix. CPAK phenotypes are defined according to the hip–knee angle (HKA) and joint line obliquity (JLO), resulting in nine alignment phenotypes (**I**–**IX**). Adapted from MacDessi et al. [9].

The Coronal Plane Alignment of the Knee (CPAK) classification describes nine knee phenotypes based on the arithmetic hip–knee–ankle angle (aHKA) and joint line obliquity (JLO) [9]. The aHKA represents constitutional coronal alignment, while JLO describes the orientation of the joint line. Combining these two parameters results in nine distinct knee phenotypes, as shown in Figure 1 [9].

Ligament balancing can be objectified by measuring knee laxity, which is defined as the medial and lateral joint space under varus and valgus stress in both extension and flexion. Differences in laxity between compartments may reflect an imbalance. Robotic-assisted TKA allows intraoperative simulation of different alignment strategies and provides objective measurements of knee laxity under standardized conditions. This enables direct comparison of ligament balances between alignment strategies within the same patient.

Previous studies have suggested that the alignment strategy influences soft tissue balance [7,10,11]. More recently, Arai et al. showed that KA resulted in a higher proportion of balanced knees compared to MA in CPAK type I patients, suggesting that the effect of a personalized alignment strategy may depend on knee phenotype [12]. However, whether similar effects are observed across other CPAK types are unknown.

The purpose of this study was to compare medial and lateral ligament laxity and overall knee balance between simulated MA and FA across different CPAK phenotypes using intraoperative robotic simulations.

## 2. Materials and Methods

### 2.1. Study Design

This is an observational cohort study including patients who underwent primary TKA using a robotic-assisted system (MAKO, Stryker, Kalamazoo, MI, USA).

Patients undergoing robotic-assisted TKA were initially considered for inclusion between July 2024 and August 2025. For patients who underwent TKA before ethics approval was obtained (approval date: 18 December 2024), radiographic and intraoperative data were retrieved after approval. For patients undergoing TKA after 18 December 2024, the data were collected prospectively during routine clinical care. Excluded were patients who underwent revision surgery, used revision components, or did not have complete radiographic data, specifically when flexion laxity measurements were missing. A total of 545 robotic-assisted TKAs were performed during the study period. Of these, 345 patients were excluded because flexion laxity measurements were missing, leaving 200 patients with complete data for the final analysis. This study was conducted in accordance with the Declaration of Helsinki and was approved by the institutional review board under reference number ME24-57 [13]. Specific informed consent for participation in this study was not obtained, as routinely collected clinical data were used in accordance with the institutional opt-out procedure. As this was an exploratory study, no formal sample size calculation was performed. Therefore, all consecutive eligible patients undergoing robotic-assisted TKA during the study period were included.

### 2.2. Surgical Technique

All procedures were performed by three orthopedic surgeons (HPVJ, DL, and MM) who have extensive experience with the use of both conventional and robotic-assisted TKAs. All procedures were performed under spinal anesthesia using a standard medial parapatellar arthrotomy. A tourniquet was not used. All procedures were performed according to the same standardized robotic-assisted surgical workflow using the MAKO system and followed the same functional alignment philosophy. After registration of the knee in the MAKO system, knee laxity was assessed in flexion and extension. Osteophytes were removed before the final assessment and implant positioning. All patients received a cementless cruciate-retaining Stryker Triathlon TKA (Stryker, Kalamazoo, MI, USA) with a standard 9 mm polyethylene insert.

### 2.3. Laxity Assessment and Simulation

Knee laxity was assessed intraoperatively using the MAKO robotic system, which allows simulation of different alignment strategies based on individual anatomy. Laxity was defined as the medial and lateral joint space under varus and valgus stress applied by the surgeon, as defined in the MAKO robotic-arm system’s simulation software (MAKO, Stryker, Kalamazoo, MI, USA).

For each patient, two alignment strategies were evaluated: a simulated MA and the final FA strategy that was implemented during surgery. Both strategies were assessed within the same patient under identical anatomical conditions, and each patient served as his or her own control, thereby minimizing the influence of patient-related confounding factors.

Mechanical alignment was simulated by positioning the components according to conventional TKA aiming for a neutral limb alignment. The tibial component was fixed at 0° varus/valgus. This approach often results in an asymmetrical flexion gap, which is typically compensated by external rotation of the femoral component and may require additional soft tissue releases to achieve coronal balance.

Functional alignment was applied intraoperatively and aimed to restore the native joint alignment through individualized positioning of both femoral and tibial components. Component position was adjusted based on patient-specific anatomy and intraoperative laxity measurements to achieve the predefined balance criteria in flexion and extension while preserving constitutional alignment as much as possible and respecting defined boundaries [5]. The femoral coronal resection angle was limited to a maximum of 3° varus and 6° valgus relative to the mechanical axis, while tibial coronal alignment was limited to a maximum of 2° valgus and 6° varus. Femoral flexion was maintained within 0–10°. This approach allows for the balancing of the knee by minimizing the need for soft tissue release and compensates for flexion gap asymmetry through femoral component rotation [5].

Using the robotic system, the MA configuration was simulated and directly compared with the final FA within the same patient under identical anatomical conditions. No soft tissue releases were performed during the simulations. Laxity measurements were recorded for both the simulated mechanical alignment and FA in extension and flexion and separately for the medial and lateral compartments.

The concept of balance was defined based on previously published criteria [7,13]. Extension balance was defined as the difference between lateral and medial extension laxity. Medial balance was defined as the difference between medial extension and medial flexion laxity. For both alignment strategies, balance was considered when the absolute difference was ≤2 mm. Overall balance was defined as the achievement of both extension and medial balance [7,12].

### 2.4. Statistical Analysis

Baseline characteristics were analyzed using descriptive statistics, with the mean ± standard deviation (SD) for continuous variables and frequencies with their respective percentages for discrete and dichotomous variables. Mean and median differences in HKA, JLO, MPTA and LDFA between native alignment and FA were compared using paired *t*-tests. The normality of the paired differences was assessed using the Shapiro–Wilk test. For differences in CPAK classification (categorical data), the Stuart–Maxwell test was used. Subgroup analyses were stratified according to native CPAK classification [9] and were considered exploratory because of the relatively small sample sizes within several CPAK subgroups. Within each native CPAK subtype, simulated mechanical and functional laxity values were compared using paired *t*-tests. Differences in the proportion of balanced knees between simulated MA and FA were analyzed using McNemar’s test. A *p*-value < 0.05 was considered statistically significant. A Bonferroni correction was applied to the three predefined primary outcomes (extension balance, medial balance, and overall balance), resulting in a significance threshold of *p* < 0.017. Statistical analyses were conducted with Statistical Package for the Social Sciences (SPSS, version 28.0.1.0 for Windows). A sensitivity analysis was performed to determine the minimum difference in overall balance between FA and MA that could be detected with the available sample size.

## 3. Results

**Figure 2:**
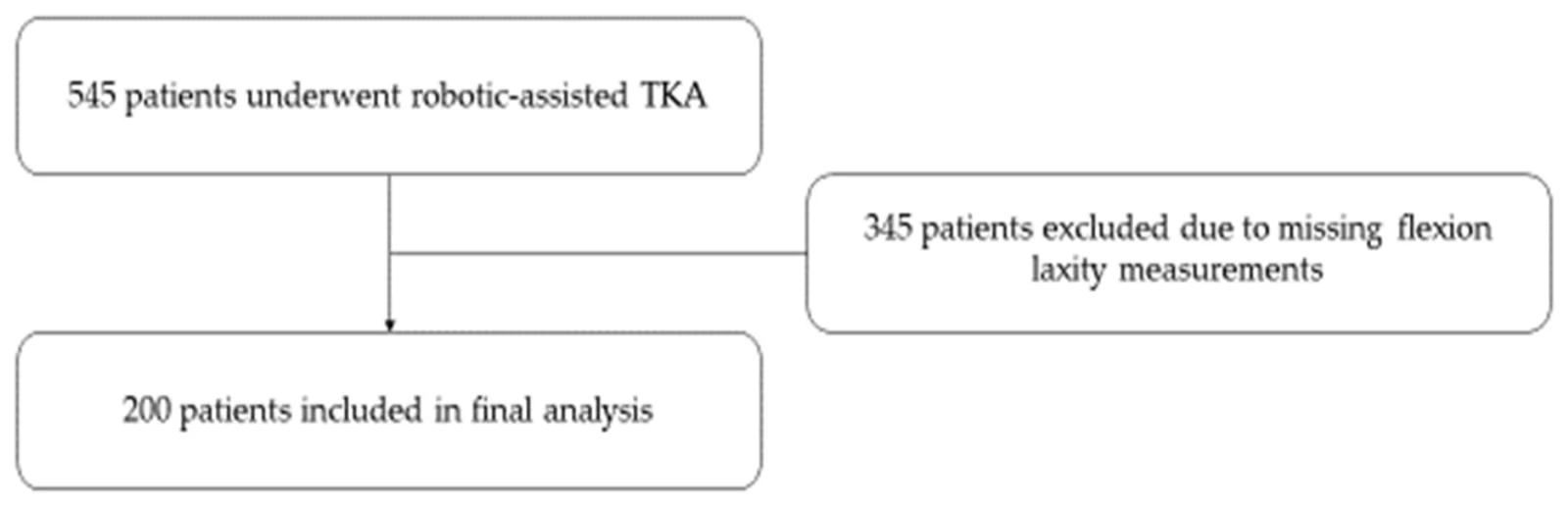
Flowchart of patient inclusion and exclusion.

A total of 545 patients underwent surgery performed by three surgeons (HPVJ, DL, and MM) between July 2024 and August 2025. Of these, 345 were excluded due to incomplete intraoperative data, leaving 200 patients for the final analysis (Figure 2).

The study population was composed of 84 (42%) women and 116 (58%) men. The mean age was 69.2 ± 14.1 years. The mean body mass index (BMI) was 29.0 ± 4.6 kg/m^2^. A total of 102 (51%) procedures were performed on the left knee and 98 (49%) were performed on the right knee. Most patients had an American Society of Anesthesiologists (ASA) score of II (53%) or III (37%), while 8% were classified as ASA I and 2% were classified as ASA IV (Table 1).

### 3.1. Alignment

Alignment parameters were significantly different between native alignment and FA (Table 2). The mean HKA was lower in FA compared to native alignment (0.10 ± 3.41° vs. 1.27 ± 3.48°, *p* < 0.001). Similarly, the mean MPTA and mean LDFA differed significantly between native alignment and FA (both *p* < 0.001). No statistically significant difference was observed for JLO (*p* = 0.139).

### 3.2. CPAK Distribution

In native alignment, the most common CPAK types were type VI (38.5%) and type VIII (30.0%), followed by type II (13.0%), as shown in Table 3. The CPAK distribution was based on native preoperative alignment.

### 3.3. Laxity

Subgroup analyses stratified by native CPAK classification showed differences in laxity between simulated MA and FA in the same patient (Table 4). CPAK phenotypes represented by fewer than 10 patients were excluded from subgroup analyses because statistical comparisons could not be performed.

In CPAK type II, extension lateral laxity was 4.4 (±1.7) mm in MA versus 1.1 (±1.5) mm in FA (*p* < 0.001). Flexion lateral laxity was 4.8 (±2.1) mm in MA versus 1.4 (±0.8) mm in FA (*p* < 0.001). Extension medial laxity was 0.6 (±0.8) mm versus −0.1 (±1.3) mm (*p* = 0.059). Flexion medial laxity was −0.1 (±1.1) mm versus 0.4 (±0.8) mm (*p* = 0.049).

In CPAK type V, extension lateral laxity was 3.0 (±2.4) mm versus 0.7 (±1.0) mm (*p* = 0.001) and flexion lateral laxity was 1.9 (±1.7) mm versus 0.6 (±0.5) mm (*p* = 0.010).

In CPAK type VI, extension lateral laxity was 2.4 (±1.6) mm versus 0.9 (±1.1) mm (*p* < 0.001) and flexion lateral laxity was 2.6 (±1.7) mm versus 0.9 (±0.7) mm (*p* < 0.001).

In CPAK type VIII, extension medial laxity was 1.4 (±1.8) mm versus −0.1 (±1.2) mm (*p* < 0.001), extension lateral laxity was 0.7 (±1.3) mm versus −0.0 (±1.2) mm (*p* = 0.002), and flexion lateral laxity was 1.4 (±2.0) mm versus 0.6 (±0.8) mm (*p* < 0.001).

In CPAK type IX, extension medial laxity, extension lateral laxity and flexion lateral laxity differed significantly between MA and FA (all *p* < 0.05). No significant difference was found for flexion medial laxity (*p* = 0.153).

### 3.4. Knee Balance

Subgroup analyses stratified by native CPAK classification showed differences in knee balance between simulated MA and FA (Table 5, Table 6 and Table 7).

In CPAK type II, extension imbalance was 3.81 ± 1.64 mm in MA versus 1.21 ± 0.85 mm in FA (*p* < 0.001), with the proportion of balanced knees being 11.5% in MA and 92.3% in FA (*p* < 0.001). In CPAK type V, the proportion of balanced knees was 55.6% in MA and 100% in FA (*p* = 0.008). In CPAK type VI, it was 64.9% and 98.7%, respectively (*p* < 0.001) (Table 5).

In CPAK type VI, medial imbalance was 1.34 ± 1.17 mm in MA versus 0.76 ± 0.67 mm in FA (*p* < 0.001). In CPAK type VIII, medial imbalance was 1.23 ± 0.93 mm in MA versus 0.76 ± 0.74 mm in FA (*p* = 0.005). After Bonferroni correction, no significant differences in medial imbalance were observed in CPAK types II (*p* = 0.091), V (*p* = 0.029), and IX (*p* = 0.901) (Table 6).

Overall balance was achieved in 50.5% of knees with simulated MA compared with 94.0% with FA (*p* < 0.001). A sensitivity analysis showed that, with a sample size of 200 patients, a difference of approximately 14 percentage points in overall balance could be detected with 80% statistical power. The observed difference was 43.5 percentage points.

**Figure 3:**
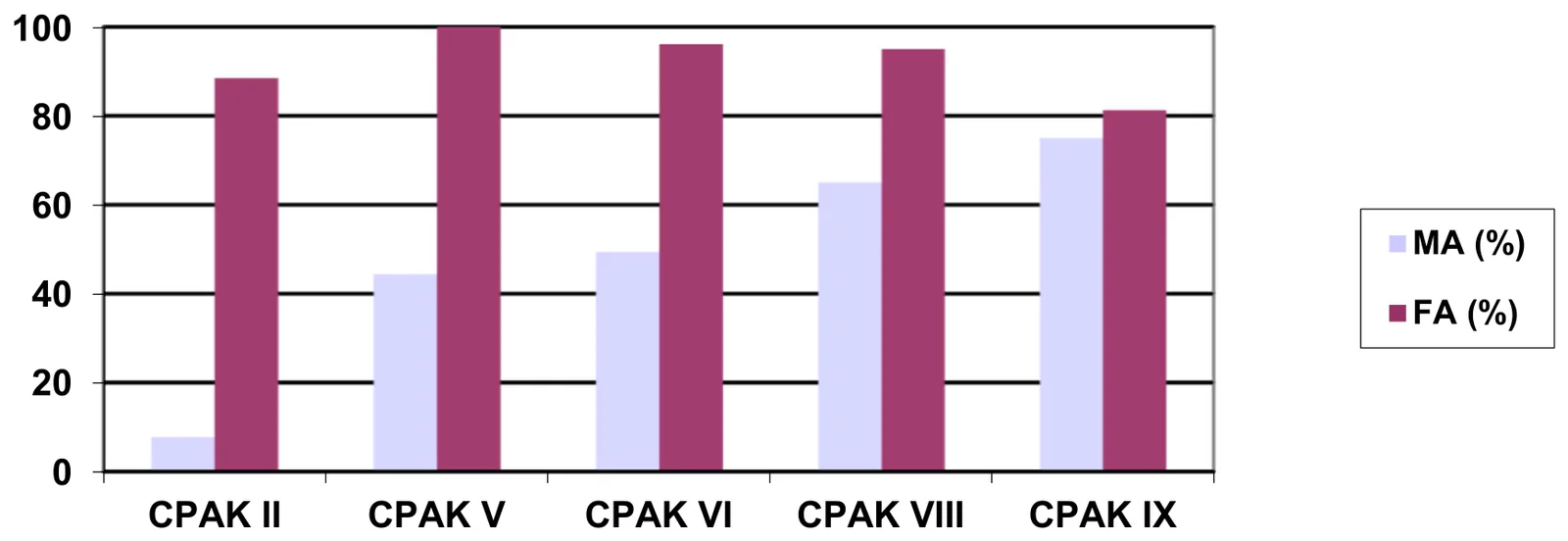
Proportion of overall balanced knees after simulated mechanical alignment (MA) and functional alignment (FA) according to native CPAK phenotype. CPAK: Coronal Plane Alignment of the Knee.

Overall balance was achieved in 7.7% versus 88.5% of knees in MA and FA in CPAK type II (*p* < 0.001), in 44.4% versus 100% in CPAK type V (*p* = 0.002), in 49.4% versus 96.1% in CPAK type VI (*p* < 0.001), and in 65.0% versus 95.0% in CPAK type VIII (*p* < 0.001). In CPAK type IX, overall balance was 75.0% in MA and 81.3% in FA (*p* = 1.000) (Table 7 and Figure 3).

## 4. Discussion

The most important finding of this study is that FA resulted in improved knee balance compared to simulated MA, with the largest effects observed in CPAK type II, while substantial improvements were also seen in CPAK types V, VI and VIII. These improvements were mainly driven by reductions in extension imbalance, resulting in a substantially higher proportion of balanced knees. In contrast, differences in medial balance were smaller and less consistent across CPAK types. These findings suggest that the effect of the alignment strategy on ligament balance is dependent on the underlying knee phenotype.

Our findings are consistent with previous studies showing that individualized alignment strategies may improve soft tissue balance compared with MA. Arai et al. reported a higher proportion of balanced knees with kinematic alignment than with MA in CPAK type I patients, particularly through improved extension balance [12]. Blakeney et al. similarly found less gap imbalances with restricted kinematic alignment compared with MA in TKA simulations [10]. Our findings support the concept that restoring native alignment may reduce the need for soft tissue releases to compensate for ligament imbalance.

In addition, recent studies have shown that alignment parameters vary across knee phenotypes. Zambianchi et al. demonstrated that femoral-component rotational alignment differs significantly between CPAK types, further supporting the concept that implant positioning should be adapted to the individual knee phenotype [14]. Together, these findings suggest that the effect of the alignment strategy on ligament balance depends on the underlying knee phenotype.

To our knowledge, this study is the first that extends these findings by including more CPAK phenotypes, demonstrating that the effect of the alignment strategy differs between knee types. The largest improvement was observed in CPAK type II, suggesting that alignment strategies that restore native alignment may be particularly relevant in knees with greater baseline imbalance. In contrast, no significant improvement was observed in CPAK type IX. A possible explanation is that knees with this phenotype already demonstrated a relatively high proportion of balanced knees after simulated MA, leaving limited room for further improvement with FA. Although FA improved intraoperative ligament balance in this study, the clinical relevance of these findings remains uncertain. It is unclear whether improvements in intraoperative ligament balance translate into better patient-reported outcomes. Future studies should evaluate this relationship.

The definition of balance used in this study should be interpreted with caution. Previous studies have demonstrated that the native knee is not completely symmetrical, with differences in medial and lateral laxity and greater lateral laxity in flexion [15,16,17]. Therefore, a smaller difference between medial and lateral laxity does not necessarily indicate a better restored native knee. In our study, the ≤2 mm threshold was used as a predefined threshold to compare simulated MA and FA, rather than as a target for complete symmetry.

Our study has several limitations. First, MA was simulated rather than performed and no soft tissue releases were applied, which may not be representative in clinical practice. However, soft tissue releases might be associated with inferior clinical outcomes in the short term and are preferably avoided [4,18,19]. In FA, knee balance is primarily achieved through implant positioning, thereby reducing the need for soft tissue releases. Second, only intraoperative laxity was assessed and no postoperative clinical outcomes were included. Although clinical outcomes were not evaluated in the present study, previous research has suggested that improved soft tissue balance may contribute to higher patient satisfaction, better knee function and prosthesis longevity after TKA [2,3]. Therefore, the substantial improvements in balance observed in CPAK types II, V and VI may have clinically relevant implications that need further investigation. The CPAK distribution in our cohort differed from that in previously reported populations, with no type I knees and a higher proportion of types VI and VIII [9,20]. Since patients were excluded due to missing flexion laxity data rather than CPAK phenotype, selection based on CPAK type is unlikely. Nevertheless, this distribution may limit the generalizability of the CPAK subgroup findings. Third, although all procedures were performed according to a standardized robotic-assisted workflow by experienced surgeons, inter-rater variability in intraoperative laxity assessment cannot be excluded. Furthermore, individual comorbidities were not collected. However, the overall preoperative health status of our study population was described using the ASA classification. Finally, some CPAK subgroups were relatively small. Therefore, the subgroup analyses should be considered exploratory. In addition, correction for multiple testing was applied to the three predefined primary outcomes. No correction was applied to the remaining CPAK subgroup analyses because these were considered exploratory. Consequently, the subgroup findings should be interpreted with caution.

Future studies should evaluate whether the observed improvements in intraoperative ligament balance also result in improved clinical outcomes. Also, further research is needed to confirm the observed differences between CPAK phenotypes and to identify which patients benefit most from individualized alignment.

## 5. Conclusions

Functional alignment was associated with improved ligament balance compared with simulated MA in robotic-assisted TKA. These improvements were observed in the overall cohort and across several native CPAK phenotypes. As the subgroup analyses were exploratory and based on relatively small sample sizes and the CPAK distribution differed from previously reported populations, our findings should be interpreted with caution. Future studies should investigate whether the observed intraoperative improvements in ligament balance translate into better clinical outcomes.

## Figures and Tables

**Table 1:** Baseline characteristics.

	Total (*n* = 200)
Mean age (years, SD)	69.2 (±14.1)
Sex (*n*, %)	
female	84 (42%)
male	116 (58%)
BMI (kg/m^2^, SD)	29.0 (±4.6)
Side (*n*, %)	
Right	98 (49%)
Left	102 (51%)
ASA-score (*n*, %)	
I	16 (8%)
II	106 (53%)
III	74 (37%)
IV	4 (2%)

ASA: American Society of Anesthesiologists, BMI: body mass index, SD: standard deviation.

**Table 2:** HKA, JLO, MPTA and LDFA for native alignment and FA.

	Native Alignment	FA *	*p*-Value
HKA (º, SD)	1.27 ± 3.48	0.10 ± 3.41	<0.001
JLO (º, SD)	173.67 ± 3.50	173.86 ± 3.44	0.139
MPTA (º, SD)	87.47 ± 2.69	86.98 ± 2.53	<0.001
LDFA (º, SD)	86.20 ± 2.23	86.88 ± 2.31	<0.001

* FA: functional alignment; HKA: hip–knee angle; JLO: joint line obliquity; MPTA: medial proximal tibial angle; LDFA: lateral distal femoral angle; SD standard deviation.

**Table 3:** Distribution of CPAK classification for native alignment.

	Native Alignment
CPAK * (*n*, %)	
I	0 (0%)
II	26 (13.0%)
III	2 (1.0%)
IV	0 (0%)
V	18 (9%)
VI	77 (38.5%)
VII	1 (0.5%)
VIII	60 (30.0%)
IX	16 (8.0%)

* CPAK: Coronal Plane Alignment of the Knee.

**Table 4:** Comparison of ligament laxity between simulated MA and FA according to native CPAK type.

CPAK *		MA	FA	*p*-Value
II (*n* = 26)	Extension lateral (mm, SD)	4.4 ± 1.7	1.1 ± 1.5	<0.001
	Extension medial (mm, SD)	0.6 ± 0.8	−0.1 ± 1.3	0.059
	Flexion lateral (mm, SD)	4.8 ± 2.1	1.4 ± 0.8	<0.001
	Flexion medial (mm, SD)	−0.1 ± 1.1	0.4 ± 0.8	0.049
V (*n* = 18)	Extension lateral (mm, SD)	3.0 ± 2.4	0.7 ± 1.0	0.001
	Extension medial (mm, SD)	1.5 ± 1.8	0.2 ± 0.9	0.017
	Flexion lateral (mm, SD)	1.9 ± 1.7	0.6 ± 0.5	0.010
	Flexion medial (mm, SD)	0.3 ± 1.9	0.3 ± 0.4	1.000
VI (*n* = 77)	Extension lateral (mm, SD)	2.4 ± 1.6	0.9 ± 1.1	<0.001
	Extension medial (mm, SD)	0.6 ± 1.2	0.3 ± 1.1	0.073
	Flexion lateral (mm, SD)	2.6 ± 1.7	0.9 ± 0.7	<0.001
	Flexion medial (mm, SD)	−0.1 ± 1.4	0.3 ± 0.4	0.005
VIII (*n* = 60)	Extension lateral (mm, SD)	0.7 ± 1.3	−0.0 ± 1.2	0.002
	Extension medial (mm, SD)	1.4 ± 1.8	−0.1 ± 1.2	<0.001
	Flexion lateral (mm, SD)	1.4 ± 2.0	0.6 ± 0.8	<0.001
	Flexion medial (mm, SD)	0.8 ± 1.9	0.4 ± 0.6	0.153
IX (*n* = 16)	Extension lateral (mm, SD)	1.6 ± 1.7	−0.0 ± 1.2	0.002
	Extension medial (mm, SD)	1.3 ± 1.5	−0.1 ± 1.2	<0.001
	Flexion lateral (mm, SD)	1.8 ± 2.1	0.6 ± 0.8	<0.001
	Flexion medial (mm, SD)	1.4 ± 1.3	0.4 ± 0.6	0.153

* CPAK: Coronal Plane Alignment of the Knee; FA: functional alignment; MA: mechanical alignment; mm: millimeter; *n*: number; SD: standard deviation.

**Table 5:** Comparison of extension balance between simulated MA and FA according to native CPAK type.

CPAK *	*n*	MA (mm, SD)	FA (mm, SD)	*p*-Value	MA Balanced	FA Balanced	*p*-Value
II	26	3.81 ± 1.64	1.21 ± 0.85	<0.001	3 (11.5%)	24 (92.3%)	<0.001
V	18	2.03 ± 1.23	0.61 ± 0.40	<0.001	10 (55.6%)	18 (100%)	0.008
VI	77	1.95 ± 1.48	0.60 ± 0.56	<0.001	50 (64.9%)	76 (98.7%)	<0.001
VIII	60	1.38 ± 1.53	0.37 ± 0.47	<0.001	47 (78.3%)	60 (100%)	<0.001
IX	16	1.28 ± 1.06	0.50 ± 0.45	0.017	13 (81.3%)	16 (100%)	0.250

* CPAK: Coronal Plane Alignment of the Knee; FA: functional alignment; MA: mechanical alignment; mm: millimeter; *n*: number; SD: standard deviation. *p*-values are unadjusted. Statistical significance for the primary outcomes was assessed using a Bonferroni-adjusted significance threshold of *p* < 0.017.

**Table 6:** Comparison of medial balance between simulated MA and FA according to native CPAK type.

CPAK *	*n*	MA (mm, SD)	FA (mm, SD)	*p*-Value	MA Balanced	FA Balanced	*p*-Value
II	26	1.29 ± 0.79	0.90 ± 0.74	0.091	23 (88.5%)	25 (96.2%)	0.625
V	18	1.56 ± 1.34	0.75 ± 0.39	0.029	13 (72.2%)	18 (100%)	0.062
VI	77	1.34 ± 1.17	0.76 ± 0.67	<0.001	61 (79.2%)	75 (97.4%)	<0.001
VIII	60	1.23 ± 0.93	0.76 ± 0.74	0.005	48 (80.0%)	57 (95.0%)	0.022
IX	16	1.16 ± 1.09	1.19 ± 1.06	0.901	14 (87.5%)	13 (81.3%)	1.000

* CPAK: Coronal Plane Alignment of the Knee; FA: functional alignment; MA: mechanical alignment; *n*: number; mm: millimeter, SD: standard deviation. *p*-values are unadjusted. Statistical significance for the primary outcomes was assessed using a Bonferroni-adjusted significance threshold of *p* < 0.017.

**Table 7:** Comparison of overall balance between simulated MA and FA according to native CPAK type.

CPAK *	*n*	MA Balanced	FA Balanced	*p*-Value
II	26	2 (7.7%)	23 (88.5%)	<0.001
V	18	8 (44.4%)	18 (100%)	0.002
VI	77	38 (49.4%)	74 (96.1%)	<0.001
VIII	60	39 (65.0%)	57 (95.0%)	<0.001
IX	16	12 (75.0%)	13 (81.3%)	1.000

* CPAK: Coronal Plane Alignment of the Knee; FA: functional alignment; MA: mechanical alignment; *n*: number. *p*-values are unadjusted. Statistical significance for the primary outcomes was assessed using a Bonferroni-adjusted significance threshold of *p* < 0.017.

## Data Availability

The data presented in this study are available from the corresponding author upon reasonable request. The data are not publicly available due to privacy restrictions.

## Abbreviations

The following abbreviations are used in this manuscript:

ASA	American Society of Anesthesiologists
BMI	Body Mass Index
CPAK	Coronal Plane Alignment of the Knee
FA	Functional Alignment
HKA	Hip–Knee Angle
JLO	Joint Line Obliquity
KA	Kinematic Alignment
LDFA	Lateral Distal Femoral Angle
MA	Mechanical Alignment
MPTA	Medial Proximal Tibial Angle
SD	Standard Deviation
SPSS	Statistical Package for the Social Sciences
TKA	Total Knee Arthroplasty

## References

[1] DeFrance M.J., Scuderi G.R. (2023). Are 20% of patients actually dissatisfied following total knee arthroplasty? A systematic review of the literature. J. Arthroplast..

[2] Hadi M., Barlow T., Ahmed I., Dunbar M., McCulloch P., Griffin D. (2016). Does malalignment affect patient reported outcomes following total knee arthroplasty: A systematic review of the literature. SpringerPlus.

[3] Rivière C., Iranpour F., Auvinet E., Howell S., Vendittoli P.A., Cobb J., Parratte S. (2017). Alignment options for total knee arthroplasty: A systematic review. Orthop. Traumatol. Surg. Res..

[4] Saber A.Y., Marappa-Ganeshan R., Mabrouk A. (2023). Robotic-Assisted Total Knee Arthroplasty. StatPearls.

[5] Shatrov J., Battelier C., Sappey-Marinier E., Gunst S., Servien E., Lustig S. (2022). Functional Alignment Philosophy in Total Knee Arthroplasty—Rationale and Technique for the Varus Morphotype Using a CT-Based Robotic Platform and Individualized Planning. SICOT-J.

[6] Barroso Rosa S., Ismailidis P., Doma K., Grant A., McEwen P., Wilkinson M., Parkinson B. (2025). Influence of Total Knee Arthroplasty Alignment on Soft-Tissue Balance and Pivot Patterns: A Randomized Controlled Trial of Kinematic Versus Mechanical Alignment. J. Arthroplast..

[7] Clark G., Steer R., Wood D. (2023). Functional alignment achieves a more balanced total knee arthroplasty than either mechanical alignment or kinematic alignment prior to soft tissue releases. Knee Surg. Sports Traumatol. Arthrosc..

[8] Howell S.M., Papadopoulos S., Kuznik K.T., Hull M.L. (2013). Accurate alignment and high function after kinematically aligned TKA performed with generic instruments. Knee Surg. Sports Traumatol. Arthrosc..

[9] MacDessi S.J., Griffiths-Jones W., Harris I.A., Bellemans J., Chen D.B. (2021). Coronal Plane Alignment of the Knee (CPAK) classification. Bone Jt. J..

[10] Blakeney W., Beaulieu Y., Kiss M.O., Rivière C., Vendittoli P.A. (2019). Less gap imbalance with restricted kinematic alignment than with mechanically aligned total knee arthroplasty: Simulations on 3-D bone models created from CT scans. Acta Orthop..

[11] Van de Graaf V.A., Chen D.B., Allom R.J., Wood J.A., MacDessi S.J. (2023). Functional alignment in total knee arthroplasty best achieves balanced gaps and minimal bone resections: An analysis comparing mechanical, kinematic and functional alignment strategies. Knee Surg. Sports Traumatol. Arthrosc..

[12] Arai N., Toyooka S., Masuda H., Kawano H., Nakagawa T. (2024). Kinematic Alignment Achieves a More Balanced Total Knee Arthroplasty Than Mechanical Alignment among CPAK Type I Patients: A Simulation Study. J. Clin. Med..

[13] World Medical Association (2013). World Medical Association Declaration of Helsinki: Ethical Principles for Medical Research Involving Human Subjects. JAMA.

[14] Zambianchi F., Bazzan G., Iorio V., Cuoghi Costantini R., Seracchioli S., Catani F. (2025). Femoral Component Rotational Alignment in Robotic-Assisted Total Knee Arthroplasty with Functional Knee Positioning Varies across Knee Phenotypes without Affecting Clinical Outcomes. Knee Surg. Sports Traumatol. Arthrosc..

[15] McEwen P., Balendra G., Doma K. (2019). Medial and lateral gap laxity differential in computer-assisted kinematic total knee arthroplasty. Bone Jt. J..

[16] Okazaki K., Miura H., Matsuda S., Takeuchi N., Mawatari T., Hashizume M., Iwamoto Y. (2006). Asymmetry of mediolateral laxity of the normal knee. J. Orthop. Sci..

[17] Yoo J.C., Ahn J.H., Sung K.S., Wang J.H., Lee S.H., Bae S.W., Ahn Y.J. (2006). Measurement and comparison of the difference in normal medial and lateral knee joint opening. Knee Surg. Sports Traumatol. Arthrosc..

[18] Naito Y., Tone S., Kobayashi G., Hasegawa M. (2025). Comparison of Clinical Outcomes and Soft Tissue Laxity Between Robot-Assisted Functionally and Mechanically Aligned Total Knee Arthroplasty. Cureus.

[19] Kayani B., Konan S., Tahmassebi J., Pietrzak J.R.T., Haddad F.S. (2018). Robotic-Arm Assisted Total Knee Arthroplasty Is Associated with Improved Early Functional Recovery and Reduced Time to Hospital Discharge Compared with Conventional Jig-Based Total Knee Arthroplasty: A Prospective Cohort Study. Bone Jt. J..

[20] Pagan C.A., Karasavvidis T., Lebrun D.G., Jang S.J., MacDessi S.J., Vigdorchik J.M. (2023). Geographic Variation in Knee Phenotypes Based on the Coronal Plane Alignment of the Knee Classification: A Systematic Review. J. Arthroplast..

